# Armitage's trend test for genome-wide association analysis: one-sided or two-sided?

**DOI:** 10.1186/1753-6561-3-s7-s37

**Published:** 2009-12-15

**Authors:** Yixin Fang, Yuanjia Wang, Nanshi Sha

**Affiliations:** 1 Department of Mathematics and Statistics, Georgia State University, 750 COE, 7th Floor, 30 Pryor Street, Atlanta, Georgia 30303, USA; 2 Department of Biostatistics, Columbia University, 722 West 168 Street, Floor 6, New York 10032, USA

## Abstract

The importance of considering confounding due to population stratification in genome-wide association analysis using case-control designs has been a source of debate. Armitage's trend test, together with some other methods developed from it, can correct for population stratification to some extent. However, there is a question whether the one-sided or the two-sided alternative hypothesis is appropriate, or to put it another way, whether examining both the one-sided and the two-sided alternative hypotheses can give more information. The dataset for Problem 1 of Genetic Analysis Workshop 16 provides us with a chance to address this question. Because it is a part of a combined sample from the North American Rheumatoid Arthritis Consortium (NARAC) and the Swedish Epidemiological Investigation of Rheumatoid Arthritis (EIRA), the results from the combined sample can be used as references. To test this aim, the last 10,000 single-nucleotide polymorphisms (SNPs) on chromosome 9, which contain the common genetic variant at the *TRAF1-C5 *locus, were examined by conducting Armitage's trend tests. Examining the two-sided alternative hypothesis shows that SNPs *rs12380341 *(*p *= 9.7 × 10^-11^) and *rs872863 *(*p *= 1.7 × 10^-15^), along with six SNPs across the *TRAF1-C5 *locus, *rs1953126*, *rs10985073*, *rs881375*, *rs3761847*, *rs10760130*, and *rs2900180 *(*p*~1 × 10^-7^), are significantly associated with anti-cyclic citrullinated peptide-positive rheumatoid arthritis. But examining the one-sided alternative hypothesis that the minor allele is positively associated with the disease shows that only those six SNPs across the *TRAF1-C5 *locus are significantly associated with the disease (*p*~1 × 10^-8^), which is consistent with the results from the combined sample of the NARAC and the EIRA.

## Background

The Genetic Analysis Workshop 16 (GAW16) rheumatoid arthritis (RA) dataset is the initial batch of whole genome-wide association study (GWAS) data for the North American Rheumatoid Arthritis Consortium (NARAC) cases (*N*_1 _= 868) and controls (*N*_0 _= 1194) after removing duplicated and contaminated samples [1]. The high-throughput genotyping technology [~550 k single-nucleotide polymorphisms (SNPs)] in the NARAC data makes it a challenge to interpret this GWAS.

One of the disadvantages of the case-control GWAS is that they are prone to a number of biases including population stratification [2]. The importance of considering confounding due to population stratification in GWAS using case-control designs [3,4] has been a source of debate. The Armitage's trend tests can correct for population stratification to some extent [5-7]; some other methods based on the Armitage's trend tests have also been developed, such as genomic control approach [8,9]. However, there is still a question as to whether the one-sided or the two-sided alternative hypothesis is appropriate, or put it another way, whether examining both the one-sided and the two-sided alternative hypotheses can give more information. The dataset for the Problem 1 of GAW16 provides us with a chance to address this question. Because it is a part of a combined sample from the NARAC and the Epidemiological Investigation of Rheumatoid Arthritis (EIRA), the results from the combined sample can be used as references.

To this aim, the last 10,000 SNPs on chromosome 9, which contains the common genetic variant at the *TRAF1-C5 *locus, were examined by conducting Armitage's trend tests. Two alternative hypotheses, the two-sided alternative hypothesis that the genotypes at a locus are associated with the disease and the one-sided alternative hypothesis that the minor allele at a locus is positively associated with the disease, were considered. Three types of scores, co-dominant score, dominant score, and recessive score, were chosen to construct the Armitage's trend tests.

## Methods

At any SNP, the data can be summarized in a contingency table as in Table 1. Always assume that "*M*" is the major allele and "*m*" is the minor allele. Scores *x*_0_, *x*_1_, and *x*_2_, are chosen to construct Armitage's trend test. The Armitage's trend test statistic is defined as [5,6].

**Table 1:** Contingency table at any SNP (*M *is major allele and m is minor allele)

	Genotype			Total
		
	*MM*	*Mm*	*Mm*	
Case	*n*_10_	*n*_11_	*n*_12_	*N*_1_
Control	*n*_00_	*n*_01_	*n*_02_	*N*_0_
Total	*N*_+0_	*N*_+1_	*N*_+2_	*N*
Score	*x*_0_	*x*_1_	*x*_2_	

Under the null hypothesis, it is approximately distributed with . This test statistic is suitable for the two-sided alternative hypothesis that the genotypes at a SNP are associated with the disease of interest. As discussed in Armitage [5], whatever the scoring system chosen, the validity of the test  is not affected, but the choice of scoring system affects the power of the test. There are three common choices of scoring system: 1) co-dominant score: *x*_0 _= 0, *x*_1 _= 1, and *x*_2 _= 2; 2) dominant score: *x*_0 _= 0, *x*_1 _= 1, and *x*_2 _= 1; 3) recessive score: *x*_0 _= 0, *x*_1 _= 0, and *x*_2 _= 1. Here, the names of scoring systems are in favor of the minor allele "*m*".

From the rationale of the genetic association analysis (see, for example, Risch and Merikangas [10]), it is more informative to look at two one-sided alternative hypotheses, i) the alternative that the minor allele is positively associated with the disease and ii) the alternative that the major allele is positively associated with the disease. Furthermore, because the disease of interest is rare, it is more reasonable to concentrate on the first alternative, despite that in practice we would do better to consider both alternatives if no prior information is available on which allele is positively associated with the disease. Another reason is that it can reduce the false-positive rate.

Hereafter, we concentrate on the alternative hypothesis that the minor allele is positively associated with the disease. To this aim, one-sided can be defined as

Under the null hypothesis, it is approximately distributed with *N*(0,1). Similarly, those three scoring systems can also be used here. It is shown in Knapp [11] that if the co-dominant scoring system is chosen, then , where *F *is the Wright's coefficient of inbreeding, and *Z *is the test statistic simply comparing the frequencies of minor allele "*m*" in the case and control groups. Here the value of *F *automatically corrects the population stratification to some extent.

## Results

For simplicity of interpretation, we only consider the last 10,000 SNPs on chromosome 9, which contain the common genetic variant at the *TRAF1-C5 *locus. The same analysis can be extended to the whole genome of approximately 550,000 SNPs.

For the two-sided alternative that the genotypes at a SNP are associated with the disease, Table 2 summarizes the LOD scores (-log_10 _p) of the test ***Z***^2^, which simply compares the frequencies of the minor allele in both groups, the Armitage's tests  with co-dominant score,  with dominant score,  with recessive score, and the Wright's coefficient of inbreeding *F*; only those SNPs with LOD > 6 are reported. The SNPs across the *TRAF1-C5 *locus are marked with asterisks.

**Table 2:** LOD values for the two-sided alternative

SNP^a^	*Z* ^2 b^	^**c**^			*F* ^d^
rs4078292	6.14	5.23	**0.91^d^**	6.96	0.1958
rs12380341	11.61	10.01	**0.36**	13.45	0.1722
rs16929545	7.22	6.71	**1.01**	7.62	0.0850
*rs1953126	7.56	7.53	5.05	5.44	*0.0037*^f^
*rs10985073	6.98	6.87	5.63	4.19	*0.0173*
*rs881375	7.64	7.63	4.81	5.71	*0.0020*
*rs3761847	7.91	7.75	5.92	5.03	*0.0230*
*rs10760130	7.42	7.30	6.03	4.40	*0.0190*
*rs2900180	8.21	8.19	5.20	6.09	*0.0022*
rs872863	15.65	14.78	**1.51**	15.11	0.0617
rs888229	6.17	5.27	**1.09**	6.10	0.1914
rs11185665	7.54	6.48	**0.32**	9.68	0.1817
rs11792145	8.58	6.53	**0.04**	12.24	0.3488

^a ^Asterisks indicates SNPs are located on *TRAF1-C5*.

^b ^*Z*^2 ^is the Chi-square test comparing the frequencies of the minor allele in the two groups

^c ^Subscripts *A*1, *A*2 and *A*3 denote test (1) with score systems 1, 2, and 3, respectively

^d ^*F *is the Wright's coefficient of inbreeding

^e ^Bold font indicates  is significantly smaller than  and .

^f ^Italic font indicates F value is smaller than 0.03.

In Table 2, those six SNPs marked with asterisks have small *F *(<0.03), and this explains why their  values in the third column, which correct for population stratification, are almost the same as *Z*^2 ^in the second column. Also, for these six SNPs,  is a bit more significant than  and , and the latter two are close to each other, which means that these SNPs are very likely co-dominant. For the other seven SNPs,  is a bit more significant than , but  is not significant at all. This shows that these SNPs are very likely recessive.

Another thing learned from Table 2 is that two SNPs, *rs12380341 *and *rs872863*, have extreme large LOD scores for *Z*^2^, , and , but surprisingly they were not reported by Plenge et al. [1], which was based on the combined sample from the NARAC and the EIRA. Are these two SNPs truly associated with the disease, or are they just false positives? Table 3 summarizes the LOD values for the one-sided alternative that the minor allele at a SNP is positively associated with the disease. Similarly, *Z*_*A*1 _is the statistic *Z*_*A *_with co-dominant score, *Z*_*A*2 _dominant score, and *Z*_*A*3 _recessive score. From Table 3, only those six SNPs marked with asterisks are significant for the one-sided alternative that the minor allele is positively associated with the disease. These results are completely consistent with the ones in Plenge et al. [1]. By consider the other one-sided alternative that the major allele is positively associated with the disease, the other seven SNPs are significant. Therefore, as discussed in the preceding section, and particularly for this dataset, it seems that it is more reasonable to consider the one-sided alternative that the minor allele is positively associated with the disease.

**Table 3:** LOD values for the one-sided alternative of the minor allele

SNP^a^	*Z* ^b^	*Z*_*A*1_^c^	*Z*_*A*2_	*Z*_*A*3_
rs4078292	0.00	0.00	0.03	0.00
rs12380341	0.00	0.00	0.11	0.00
rs16929545	0.00	0.00	0.02	0.00
*rs1953126	7.86	7.83	5.35	5.74
*rs10985073	7.28	7.17	5.93	4.49
*rs881375	7.95	7.93	5.12	6.01
*rs3761847	8.21	8.05	6.22	5.33
*rs10760130	7.72	7.60	6.33	4.70
*rs2900180	8.51	8.49	5.50	6.39
rs872863	0.00	0.00	0.01	0.00
rs888229	0.00	0.00	0.02	0.00
rs11185665	0.00	0.00	0.63	0.00
rs11792145	0.00	0.00	0.27	0.00

^a ^Asterisks indicates SNPs are located on *TRAF1-C5*.

^b ^*Z *is the *z*-test comparing the frequencies of the minor allele in the two groups

^c ^Superscripts *A*1, *A*2 and *A*3 denote the test (2) with score systems 1, 2, and 3, respectively

## Discussion

The question of whether the two-sided alternative or the one-sided alternatives should be considered is intractable, but this manuscript attempts to raise the question and address it to some extent. Table 3 shows that if we concentrate on the one-sided alternative that the minor allele is positively associated with the disease, we get exactly the same results as Plenge et al. [1]. For rare diseases, and we have reason to believe that the alleles positively associated with them have low frequencies in a general population. Based on this belief (or alternative hypothesis), it seems that those SNPs without asterisks are false positives under the two-sided alternative.

But if we do not want to believe that the minor allele is positively associated with the disease and do not want to miss any SNPs related to the disease, we had better consider the two-sided alternative.

## Conclusion

More information can be gained from GWAS by using multiple scoring systems in the Armitage's trend tests and examining both the one-sided and the two-sided alternative hypotheses.

## List of abbreviations used

EIRA: Epidemiological Investigation of Rheumatoid Arthritis; GAW16: Genetic Analysis Workshop 16; GWAS: Genome-wide association; NARAC: North American Rheumatoid Arthritis Consortium; RA: Rheumatoid arthritis; SNP: Single-nucleotide polymorphism(s).

## Competing interests

The authors declare that they have no competing interests.

## Authors' contributions

YF conceived the idea, performed the statistical analysis, and drafted the manuscript. YW and NS helped to perform the statistical analysis and draft the manuscript. All authors read and approved the final manuscript.
